# Performance of Different Scan Protocols of Fetal Echocardiography in the Diagnosis of Fetal Congenital Heart Disease: A Systematic Review and Meta-Analysis

**DOI:** 10.1371/journal.pone.0065484

**Published:** 2013-06-04

**Authors:** Yifei Li, Yimin Hua, Jie Fang, Chuan Wang, Lina Qiao, Chaomin Wan, Dezhi Mu, Kaiyu Zhou

**Affiliations:** 1 Department of Pediatric Cardiovascular Disease, West China Second University Hospital, Sichuan University, Chengdu, Sichuan, China; 2 Ministry of Education Key Laboratory of Women and Children’s Diseases and Birth Defects, West China Second University Hospital, Sichuan University, Chengdu, Sichuan, China; 3 West China Medical School of Sichuan University, Chengdu, Sichuan, China; 4 Program for Changjiang Scholars and Innovative Research Team in University, West China Second University Hospital, Sichuan University, Chengdu, Sichuan, China; 5 State Key Laboratory of Oral Disease, West China Hospital of Stomatology, Sichuan University, Chengdu, Sichuan, China; University of Adelaide, Australia

## Abstract

**Objective:**

The rapid progress in fetal echocardiography has lead to early detection of congenital heart diseases. Increasing evidences have shown that prenatal diagnosis could be life saving in certain cases. However, there is no agreement on which protocol is most adaptive diagnostic one. Thus, we use meta-analysis to conduct a pooled performance test on 5 diagnostic protocols.

**Methods:**

We searched PUBMED, EMBASE, the Cochrane Central Register of Controlled Trials and WHO clinical trails registry center to identify relevant studies up to August, 2012. We performed meta-analysis in a fixed/random-effect model using Meta-disc 1.4. We used STATA 11.0 to estimate the publication bias and SPSS 17.0 to evaluate variance.

**Results:**

We use results from 81 studies in 63 articles to analyze the pooled accuracy. The overall performance of pooled sensitivities of spatiotemporal image correlation (STIC), extend cardiac echography examination (ECEE) and 4 chambers view + outflow tract view + 3 vessels and trachea view (4 CV+OTV+3 VTV) were around 0.90, which was significant higher than that of 4 chambers view + outflow tract view or 3 vessels and trachea view (4 CV+OTV/3 VTV) and 4 chambers view (4 CV). Unfortunately the pooled specificity of STIC was 0.92, which was significant lower than that of other 4 protocols which reached at 1.00. The area under the summary receiver operating characteristic curves value of STIC, ECEE, 4 CV+OTV+3 VTV, 4 CV+OTV/3 VTV and 4 CV were 0.9700, 0.9971, 0.9983, 0.9929 and 0.9928 respectively.

**Conclusion:**

These results suggest a great diagnostic potential for fetal echocardiography detection as a reliable method of fetal congenital heart disease. But at least 3 sections view (4 CV, OTV and 3 VTV) should be included in scan protocol, while the STIC can be used to provide more information for local details of defects, and can not be used to make a definite diagnosis alone with its low specificity.

## Introduction

Congenital heart disease (CHD) is the most common birth abnormality, with a incidence of 6–8‰ in all live births [1]. 20% of those who survive have major CHD. Many of them need surgical procedure in early life stage to retain their life [2]. In certain cases of fetal cardiac and other structural anomalies, prenatal diagnosis may be helpful or even life saving [3]–[5], with prenatal diagnosis providing optimal perinatal and perioperative management [6]. Fortunately, constant advance in ultrasound imaging has improved the imaging quality and the accuracy of earlier detection [7], [8]. At first, 4 chambers view (4 CV) was used to scan fetal heart defects, then outflow tract view (OTV) and 3 vessels trachea view (3 VTV) were added to increase accuracy of fetal echocardiography. Nowadays, extend cardiac echography examination (ECEE) was carried out as a specific protocol to identify some minimal defects in utero and provide more detail information on suspicious fetal heart. Since spatiotemporal image correlation (STIC), was first introduced for fetal echocardiography in 2003 [9]. Many studies have described its application to scanning normal and anomalous fetal hearts [10], [11]. Also cardiovascular diseases can be diagnosed by assessing abnormal flow behavior in the heart using noninvasive assessment based on magnetic resonance. And with the computer-aided flow analysis, high quailty image can be catched to make a reliable diagnosis during fetal life [12]–[15]. Compared to ultrasound diagnostic protocols, the magnetic resonance examination must be performed in hospital and spend a longer time as well as its higher cost. So the echocaridiography is still the most popular scan method and perfomed in many kinds of examination during pregnancy.

So far, a lot of studies have demonstrated the short-term and long-term prognostic benefit resulting from the prenatal diagnosis of CHD. Nowadays, 4 CV, 4 CV+OTV/3 VTV, 4 CV+OTV+3 VTV, ECEE and STIC were the most popular scan protocols for fetal CHD diagnosis during last several decades [8], [16], [17]. However, Moreover, no general agreement has been recognized on how to choose from the 5 protocols for fetal CHD diagnosis, even though some comparison studies have been done on the accuracy among different scan protocols. Thus, in the meta-analysis, we estimated the accuracy of fetal diagnosis and compared sensitivities and specificities among 5 diagnostic protocols.

## Materials and Methods

### Study Protocol

This analysis was conducted in accordance with a predetermined protocol following the recommendations of Deeks et al. [18]. And there is no existed protocol. The data collection and reporting were in accordance with Preferred Reporting Items for Systematic Reviews and Meta-Analyses: The PRISMA Statement (Table S1).

### Search Strategy

Pubmed, Embase, the Cochrane Central Register of Controlled Trials and World Health Organization clinical trails registry center were searched using a high sensitive and high specific search strategy,which was “diagnosis AND (heart defects, congenital [MeSH Terms] OR congenital heart disease) AND (ultrasonography OR sonography OR echocardiography OR ultrasound) AND (prenatal OR antenatal OR intrauterine OR in utero)”. Search was updated to August 2012. The language restriction was used only for English published papers.

### Study Selection

Citations initially selected by systematic search were first retrieved as title and/or abstract and preliminarily screened. Potentially relevant reports were then retrieved as complete manuscripts and assessed for compliance to inclusion and exclusion criteria.

The inclusion criteria were as followings: 1) the patients were taken fetal echocardiography or ultrasound examination in utero; 2) diagnostic test; 3) the prenatal diagnosis confirmed by neonatal echocardiography or autopsy or surgery or cardiac catheterization; 4) contained the date of true positive, false positive, false negative and true negative; or the sensitivity, specificity and essential sample size.

The exclusion criteria were as followings: 1) the total sample size was quite small (total sample size ≤15); 2) the same cohort had been studied in other study; 3) unable to construct 2×2 table; 4) special echocardiography use for diagnosis; 5) not focused on CHD; 6) conferences articles.

### Data Collection and Assessment of Study Quality

Two investigators (Yifei Li, Jie Fang) independently assessed eligibility of reports at the title and/or at abstract level, with a third reviewer (Kaiyu Zhou) determining the divergences together; studies that met the inclusion criteria were selected for further analysis.

The quality of each study’s methodology was assessed using the 14-item Quality Assessment of Diagnostic Accuracy Studies (QUADAS) list [19]. Each question was assigned with a response of yes, no, or unclear when evaluating each of the included studies. Since the assessment of quality related strongly to the reporting of results, a well conducted study could score poorly if the methods and results were not reported in sufficient detail. Therefore, we did not report the assessment in scores but in descriptive forms only.

### Publication Bias

Publication bias was tested using funnel plots and the Deek’s test by Stata statistical software (STATA) version 11.0. An asymmetric distribution of data points in the funnel plot and a quantified result of P, 0.10 in the Deek’s test indicated the presence of potential publication bias [20].

### Heterogeneity

The Χ^2^ test was used to examine heterogeneity in pooling sensitivity and specificity. The Cochran Q test was used to examine heterogeneity in pooling diagnostic odds ratio. Heterogeneity was considered to be statistically significant when P< 0.05 in these qualitative tests. The I^2^ test was also conducted in every pooling analysis to quantitatively estimate the proportion of total variation across studies that was attributable to heterogeneity rather than chance. The I^2^ value would range from 0 to 100%, with a value over 50% indicating significant heterogeneity. The existence of a threshold effect would manifest as a curvilinear shape in the summary receiver operating characteristic curves.

### Sensitivity Analysis

To determine whether any single study was incurring undue weight in the analysis, one set of study data were systematically removed, and the pooled results for the remaining studies were rechecked whether the results had a significant change. The sensitivity analysis was conducted for every study.

### Statistical Analysis

Data were analyzed using Meta-Disc Version 1.4 [21] and STATA version 11.0. The test performance of different types of echocardiography detection for the fetal CHDs was measured by the following indicators: sensitivity, specificity and diagnostic odds ratio. Sensitivity was represented by the proportion of fetus with heart malformation that was correctly identified by the positive results of different types of echocardiography. Specificity was represented by the non-heart malformation cases that were correctly identified by the negative results of different types of echocardiography. Moreover, it was more reliable to define the summary of test performance using diagnostic odds ratio than simply pooling sensitivity and specificity together across the studies. Diagnostic odds ratio was an independent indicator ranging from 0 to infinity, which represented how much greater the odds of having fetal congenital heart disease were for patient with a positive detecting result than for patient with a negative ultrasound result. The higher the diagnostic odds ratio, the better the discriminatory ability of the test was [22]. The summary receiver operating characteristic curve was plotted based on the combination of sensitivity and specificity, and the area under the curve value was then calculated as a global measurement of test performance. The closer the the area under the curve value was to 1, the better the test performance [23]. And the Χ^2^ test of evaluating the sensitivities and specificities among different types of echocardiography were performed using Statistical Product and Service Solutions (SPSS) 17.0. For all tests, a P value <0.05 was considered with significant difference. Because of potential heterogeneity between studies, effect sizes were pooled by random-effects models of DerSimonian and Laird in Meta Disc [24]. Empty cells were handled using a 0.5 continuity correction.

## Results

### Study Evaluation

**Figure 1:**
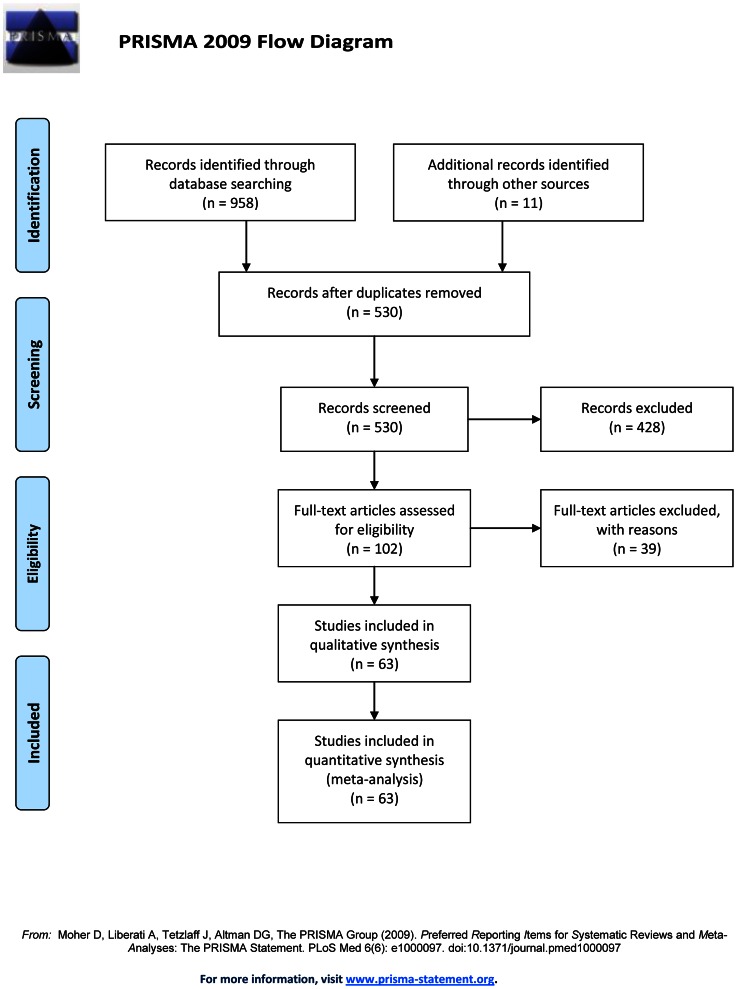
Flow diagram of study selection process.

A total of 519 citations were retrieved by the method aforementioned. After reading titles and abstracts, 428 citations were excluded according to the selection criteria, and identified the initially 91 articles. Among them, 39 articles were excluded by reading the completed articles [9], [25]–[62], in which 17 articles were unable to construct 2×2 table, 13 articles were not about diagnostic tests, 4 articles focused special echocardiography use for diagnosis, 2 articles only provided technique successful rate, 1 article didn’t focus on CHDs, 1 article was a repeated sample and 1 article was a review. Then, 11 articles were added through manual retrospective research after reading related publications [53], [57], [61], [63]–[70]. At last 63 articles with 81 diagnostic test studies for fetal CHD diagnosis were enrolled into this meta-analysis [11], [17], [63]–[123] (Figure 1). Among these 81 researches, 8 studies were about STIC, 24 studies were about ECEE, 9 studies were about 4 CV+OTV+3 VTV, 13 studies were about 4 CV+OTV or 4 CV+3 VTV and 24 studies were about 4 CV. Moreover, 16 articles contained 2 studies for such accuracy evaluation [71], [74], [76]–[79], [83], [87]–[89], [100], [101], [106], [113], [116], [119], and 1 article contained 3 studies of such accuracy evaluation [68]. The basic characteristics of included studies were showed in Table 1.

**Table 1:** Characteristics of included studies.

No.	Author	Year	Journal	Design	Countries	Sections	Types of CHDs	High/Low risk	Gestation weeks	Adequate reference standard	Fetus
1a	Volpe	2012	J Ultrasound Med	Retrospective & consecutive	Italy	4 CV+OTV+3 VTV	Unselected	Unselected	Early (11–14)	Postnatal ECHO or PM Autopsy	870
1b	Volpe	2012	J Ultrasound Med	Retrospective & consecutive	Italy	4 CV+OTV+3 VTV	Unselected	Unselected	Middle (18–22)	Postnatal ECHO or PM Autopsy	870
2	Yagel	2011	Ultrasound Obstet Gynecol	Retrospective & consecutive	Israel	ECEE	Unselected	Unselected	Early and Middle (14–16) and (22–24)	Postnatal ECHO or PM Autopsy	13101
3	Ozkutlu	2010	Anadolu Kardiyol Derg	Retrospective & consecutive	Turkey	4 CV	Unselected	Unselected	Early and Middle	Partial postnatal ECHO or PM Autopsy	1370
4	Espinoza	2010	J Ultrasound Med	Retrospective & nonconsecutive	USA+Italy+Israel+Chile	STIC	Unselected	Unselected	Middle (18–26)	Postnatal ECHO or PM Autopsy	90
5a	Bennasar	2010	Ultrasound Obstet ynecol	Prospective & consecutive	Spain	ECEE	Unselected	Unselected	Early and Middle (11–16)	Postnatal ECHO or PM Autopsy	342
5b	Bennasar	2010	Ultrasound Obstet Gynecol	Prospective & consecutive	Spain	STIC	Unselected	Unselected	Early and Middle (11–17)	Postnatal ECHO or PM Autopsy	335
6	Abu-Rustum	2010	J Ultrasound Med	Retrospective & consecutive	Lebanon	4 CV+OTV+3 VTV	Major CHDs	Unselected	Early and Middle	Postnatal ECHO	1370
7a	Wu	2009	J Ultrasound Med	Prospective & consecutive	China	4 CV+OTV+3 VTV	Unselected	Unselected	Middle (20–24)	Postnatal ECHO or PM Autopsy	8025
7b	Wu	2009	J Ultrasound Med	Prospective & consecutive	China	4 CV	Unselected	Unselected	Middle (20–24)	Postnatal ECHO or PM Autopsy	8025
8a	Bernard	2009	Ultrasound Obstet Gynecol	Retrospective & nonconsecutive	USA	4 CV	Unselected	High Risk	Middle (Mean 19)	Postnatal ECHO	117
8b	Bernard	2009	Ultrasound Obstet Gynecol	Retrospective & nonconsecutive	USA	4 CV	Unselected	High Risk	Middle (Mean 23)	Postnatal ECHO	117
9a	Bennasar	2009	Ultrasound Obstet Gynecol	Prospective & consecutive	Spain	STIC	Unselected	Unselected	Early (11–14)	Postnatal ECHO or PM Autopsy	64
9b	Bennasar	2009	Ultrasound Obstet Gynecol	Prospective & consecutive	Spain	ECEE	Unselected	Unselected	Early (11–15)	Postnatal ECHO or PM Autopsy	64
10	Paladini	2008	Ultrasound Obstet Gynecol	Prospective & consecutive	Italy	STIC	Unselected	Unselected	Middle (20)	Postnatal ECHO or PM Autopsy	364
11a	Rizzo^a^	2008	Fetal Diagn Ther	Retrospective & consecutive	Italy	STIC	Unselected	Low Risk	Middle (20.4)	Postnatal ECHO or PM Autopsy	111
11b	Rizzo^a^	2008	Fetal Diagn Ther	Retrospective & consecutive	Italy	STIC	Unselected	Low Risk	Middle (20.4)	Postnatal ECHO or PM Autopsy	111
12	Khoo	2008	Aust N Z J Obstet Gynaecol	Retrospective & consecutive	Australia	ECEE	Unselected	Unselected	Middle (>20)	Postnatal ECHO or PM Autopsy	310
13	Plesinac	2007	Int J Fertil Womens Med	Prospective & consecutive	Serbia	ECEE	Unselected	High Risk	Not provided	Postnatal ECHO or Surgery or PM Autopsy	517
14a	Pascal	2007	Cardiol Young	Retrospective & consecutive	UK	ECEE	Ventricular septal defects	Unselected	Middle and Late (18–34)	Postnatal ECHO or PM Autopsy	57
14b	Pascal	2007	Cardiol Young	Retrospective & consecutive	UK	ECEE	Coarctation of the aorta	Unselected	Middle and Late (18–34)	Postnatal ECHO or PM Autopsy	54
15	Li	2007	Chin Med J (Engl)	Retrospective & consecutive	China	ECEE	Twins in CHDs	Unselected	Middle and Late (20–37)	Postnatal ECHO or PM Autopsy	1103
16	Bakiler^b^	2007	Fetal Diagn Ther	Retrospective & consecutive	Turkey	ECEE	Unselected	High Risk	Middle (26.4)	Postnatal ECHO or PM Autopsy	197
17	Tegnander	2006	Ultrasound Obstet Gynecol	Prospective & consecutive	Norway	4 CV+3 VTV	Major CHDs	Unselected	Middle (16–22)	Postnatal ECHO or PM Autopsy	29460
18	Ogge	2006	Ultrasound Obstet Gynecol	Prospective & consecutive	Italy	4 CV+OTV	Unselected	Low Risk	Middle (16–22)	Postnatal ECHO or PM Autopsy	9074
19	Goncalves^c^	2006	J Perinat Med	Retrospective & consecutive	USA	STIC	Unselected	Unselected	Early to Late (14–41)	Postnatal ECHO or PM Autopsy	168
20a	Del Bianco	2006	J Perinat Med	Retrospective & consecutive	Italy	4 CV	Unselected	Low Risk	Middle (20–24)	Postnatal ECHO or PM Autopsy	2847
20b	Del Bianco	2006	J Perinat Med	Retrospective & consecutive	Italy	4 CV+3 VTV	Unselected	Low Risk	Middle (20–24)	Postnatal ECHO or PM Autopsy	2847
21a	Becker	2006	Ultrasound Obstet Gynecol	Prospective & consecutive	Germany	ECEE	Unselected	Low Risk	Early (11–13)	Postnatal ECHO	3094
21b	Becker	2006	Ultrasound Obstet Gynecol	Prospective & consecutive	Germany	ECEE	Unselected	High Risk	Early (11–13)	Postnatal ECHO	306
22a	Zhou	2005	Chin Med J (Engl)	Prospective & consecutive	China	4 CV	Unselected	High Risk	Early and Middle (11–16)	Postnatal ECHO or PM Autopsy	383
22b	Zhou	2005	Chin Med J (Engl)	Prospective & consecutive	China	ECEE	Unselected	High Risk	Early and Middle (11–16)	Postnatal ECHO or PM Autopsy	383
23	Sklansky^d^	2005	Ultrasound Obstet Gynecol	Retrospective & nonconsecutive	USA	STIC	Unselected	Unselected	Middle (26–28)	Fetal ECHO by 4 Reviewers	18
24	Paladini	2005	Prenat Diagn	Retrospective & consecutive	Italy	4 CV+OTV+3 VTV	Multiple pregnancies in CHDs	Unselected	Middle and Late (16–35)	Postnatal ECHO or PM Autopsy	678
25	Ozkutlu	2005	Turk J Pediatr	Prospective & consecutive	Turkey	ECEE	Unselected	High Risk	Middle and Late (18–39)	Postnatal ECHO or Cardiac catheterization or PM Autopsy	642
26	McAuliffe	2005	Am J Obstet Gynecol	Retrospective & Prospective & consecutive	Canada	4 CV+3 VTV	Unselected	High Risk	Early and Middle (11–15)	Postnatal ECHO or PM Autopsy	153
27	Machlitt	2004	Ultrasound Obstet Gynecol	Retrospective & Prospective & consecutive	Germany	4 CV	AVSD	Unselected	Middle (18–23)	Postnatal ECHO or PM Autopsy	152
28	Carvalho	2004	Heart	Retrospective & consecutive	UK	4 CV+OTV+3 VTV	Major CHDs	High Risk	Early (<16)	Postnatal ECHO or PM Autopsy	230
29	Galindo	2003	J Matern Fetal Neonatal Med	Retrospective & consecutive	Spain	4 CV+OTV+3 VTV	Unselected	High Risk	Middle (18–22)	Postnatal ECHO or PM Autopsy	138
30	Bronshtein	2003	Am J Cardiol	Retrospective & nonconsecutive	Israel	ECEE	AVSD	High Risk	Early (11–14)	Postnatal ECHO or PM Autopsy	803
31a	Weiner	2002	J Ultrasound Med	Retrospective & consecutive	Israel	4 CV+3 VTV	Unselected	High Risk	Early (11–14)	Postnatal ECHO or PM Autopsy	392
31b	Weiner	2002	J Ultrasound Med	Retrospective & consecutive	Israel	ECEE	Unselected	High Risk	Early (15–16)	Postnatal ECHO or PM Autopsy	438
31c	Weiner	2002	J Ultrasound Med	Retrospective & consecutive	Israel	ECEE	Unselected	High Risk	Middle (22–24)	Postnatal ECHO or PM Autopsy	777
32	Skeels	2002	Pediatr Cardiol	Retrospective & consecutive	USA	ECEE	Unselected	Unselected	Middle (mean 21)	Late perinatal ECHO or postnatal ECHO	614
33	Haak	2002	Ultrasound Obstet Gynecol	Prospective & consecutive	Netherlands	ECEE	Unselected	High Risk	Early (11–14)	Postnatal ECHO or PM Autopsy	38
34	Comas Gabriel	2002	Prenat Diagn	Retrospective & consecutive	Spain	4 CV+3 VTV	Unselected	High Risk	Early and Middle (12–17)	Postnatal ECHO or PM Autopsy	334
35	Meyer–Wittkopf	2001	Ultrasound Obstet Gynecol	Retrospective & consecutive	UK	ECEE	Major CHDs	High Risk	Middle and Late (17–38)	Postnatal ECHO or PM Autopsy	1037
36a	Berghella	2001	Fetal Diagn Ther	Retrospective & consecutive	USA	4 CV+OTV+3 VTV	Unselected	Unselected	Middle and Late (Mean 30.4)	Postnatal ECHO or Surgery or PM Autopsy	619
36b	Berghella	2001	Fetal Diagn Ther	Retrospective & consecutive	USA	4 CV+OTV+3 VTV	Unselected	Unselected	Middle and Late (Mean 29.4)	Postnatal ECHO or Surgery or PM Autopsy	2147
37	Simpsom	2000	BJOG	Retrospective & consecutive	UK	4 CV	Major CHDs	High Risk	Early (11–15)	Late perinatal ECHO or postnatal ECHO	226
38	Rustico	2000	Ultrasound Obstet Gynecol	Prospective & consecutive	Italy	4 CV	Major CHDs	Unselected	Early (11–14)	Late perinatal ECHO or PM Autopsy	4716
39	Zosmer	1999	Br J Obstet Gynaecol	Prospective & consecutive	UK	4 CV+OTV	Major CHDs	High Risk	Early (11–14)	Late perinatal ECHO or postnatal ECHO or PM Autopsy	398
40	Stefos	1999	J Matern Fetal Med	Prospective & consecutive	Greece	4 CV	Unselected	Unselected	Middle (18–22)	Postnatal ECHO or PM Autopsy	7236
41a	Ozkutlu	1999	Turk J Pediatr	Prospective & consecutive	Turkey	4 CV+OTV	Major CHDs	Unselected	Middle and Late (15–37)	Postnatal ECHO or Cardiac catheterization	128
41b	Ozkutlu	1999	Turk J Pediatr	Prospective & consecutive	Turkey	4 CV+OTV	Minor CHDs	Unselected	Middle and Late (15–37)	Postnatal ECHO or Cardiac catheterization	128
42a	Buskens	1996	Circulation	Prospective & consecutive	Netherlands	4 CV	Unselected	Unselected	Middle (16–24)	Postnatal ECHO or PM Autopsy	5319
42b	Buskens	1996	Circulation	Prospective & consecutive	Netherlands	4 CV	Major CHDs	Unselected	Middle (16–24)	Postnatal ECHO or PM Autopsy	5319
43	Hafner	1998	Prenat Diagn	Retrospective & consecutive	Austria	4 CV+OTV	Unselected	Low Risk	Early and Middle (10–24)	Postnatal ECHO or PM Autopsy	6541
44	Todros	1997	Prenat Diagn	Prospective & consecutive	Italy	4 CV	Unselected	Low Risk	Middle (19–22)	Postnatal ECHO or PM Autopsy	8299
45	Kirk	1997	Obstet Gynecol	Retrospective & consecutive	USA	4 CV+OTV	Unselected	Unselected	Middle and Late (>14)	Postnatal ECHO or PM Autopsy	16121
46	Crane	1997	Ultrasound Obstet Gynecol	Retrospective & Prospective & consecutive	Canada	4 CV	Unselected	Unselected	Middle and Late (16–40)	Postnatal ECHO or Surgery or PM Autopsy	409
47	Stumpflen	1996	Lancet	Retrospective & consecutive	Austria	4 CV+OTV	Unselected	Unselected	Middle (18–28)	Postnatal ECHO or PM Autopsy	3085
48	Buskens	1996	Obstet Gynecol	Retrospective & consecutive	Netherlands	ECEE	Unselected	High Risk	Middle (16–25)	Postnatal ECHO or PM Autopsy	3223
49	Saxena	1995	Indian J Pediatr	Retrospective & consecutive	Indian	4 CV	Unselected	High Risk	Middle and Late (>20)	Postnatal ECHO or PM Autopsy	993
50	Rustico	1995	Ultrasound Obstet Gynecol	Retrospective & consecutive	Italy	4 CV	Unselected	Low Risk	Middle (20–22)	Postnatal ECHO or PM Autopsy	7024
51a	Ott	1995	Am J Obstet Gynecol	Prospective & consecutive	USA	4 CV+OTV	Unselected	High Risk	Middle and Late (>15)	Postnatal ECHO	886
51b	Ott	1995	Am J Obstet Gynecol	Prospective & consecutive	USA	4 CV+OTV	Unselected	Low Risk	Middle and Late (>15)	Postnatal ECHO	1136
52	Giancotti	1995	Clin Exp Obstet Gynecol	Retrospective & consecutive	Italy	ECEE	Unselected	High Risk	Middle and Late (16–40)	Postnatal ECHO or PM Autopsy	736
53	Edwards	1995	Ultrasound Obstet Gynecol	Retrospective & consecutive	USA	ECEE	Twins in CHDs	Unselected	Middle (16–20)	Postnatal ECHO or PM Autopsy	490
54	Wilson	1994	N Z Med J	Retrospective & consecutive	New Zealand	4 CV	Unselected	High Risk	Middle (Mean 24)	Postnatal ECHO or PM Autopsy	130
55	Achiron	1994	J Ultrasound Med	Retrospective & consecutive	Israel	ECEE	Unselected	Low Risk	Early (13–15)	Postnatal ECHO or PM Autopsy	660
56	Vergani	1992	Am J Obstet Gynecol	Prospective & consecutive	Italy	4 CV	Unselected	Unselected	Middle (18–20)	Postnatal ECHO	9016
57a	Achiron	1992	BMJ	Retrospective & consecutive	Israel	4 CV	Unselected	Low Risk	Middle (18–24)	Postnatal ECHO or PM Autopsy	5347
57b	Achiron	1992	BMJ	Retrospective & consecutive	Israel	ECEE	Unselected	Low Risk	Middle (18–24)	Postnatal ECHO or PM Autopsy	5347
58	Levi	1991	Ultrasound Obstet Gynecol	Prospective & consecutive	Belgium	4 CV	Unselected	Low Risk	Middle (16–20)	Postnatal ECHO	16361
59	Martin	1990	J Am Soc Echocardiogr	Retrospective & consecutive	USA	4 CV	Unselected	High Risk	Middle (Mean 24)	Postnatal ECHO or PM Autopsy	382
60	Allan	1989	Int J Cardiol	Retrospective & consecutive	UK	ECEE	Unselected	High Risk	Middle and Late (20–34)	Postnatal ECHO or PM Autopsy	978
61	Copel	1987	Am J Obstet Gynecol	Retrospective & consecutive	USA	4 CV	Unselected	Unselected	Not provided	Postnatal ECHO	1012
62	Sholler^d^	1986	Med J Aust	Retrospective & consecutive	Australia	4 CV	Unselected	High Risk	Middle and Late (18–38)	Postnatal ECHO	36
63	Nimrod^d^	1984	Am J Obstet Gynecol	Retrospective & consecutive	Canada	4 CV	Unselected	High Risk	Middle and Late (18–36)	Postnatal ECHO	27

a Two examiners repeated the diagnostic test.

b False positive is mainly about ASD 8 cases.

c Use M-model and color doppler together.

d With small sample size.

STIC, spatiotemporal image correlation; ECEE, extended cardiac echography examination; 4 CV, 4 chamber view; OTV, outflow tract view; VTV, three-vessel trachea view; ECHO, echocardiography; PM, postmortem examination.

### Study Quality

The QUADAS list of questions was used to review the test quality of the included studies. Most of the studies satisfied a majority of the items on the QUADAS list. The most common missing items in the studies included in this analysis were reports of uninterruptible test results and withdrawn cases. In addition, almost all of the studies failed to mention the blinded interpretations between the fetal ultrasound results and the neonatal or autopsy evaluation (Table S2).

### Publication Bias

Funnel plots were used to evaluate the publication bias of included studies. Each dot represents a study and the distance between each dot and the vertical line suggests bias in each study. The absence of any asymmetric distribution suggested there was no publication bias. While the asymmetric distribution existed, that indicated that publication bias was existed. The Deek’s test revealed the possibility of significant publication bias among the included reports of ECEE (p = 0.01, 95% CI, −54.69 to −7.64) and 4 CV (p = 0.00, 95% CI, −52.92 to −17.20) evaluation pooled results. The funnel plot in Figure S2 and S5 also presented a certain degree of asymmetry, indicating the potential for publication bias among the studies included in this analysis. Otherwise, there were no significant publication bias among the included reports of STIC (p = 0.28, 95% CI, −13.03 to 37.69), 4 CV+OTV+3 VTV (p = 0.21, 95% CI, −93.30 to 24.50) and 4 CV+OTV/3 VTV (p = 0.15, 95% CI, −70.08 to 11.95) evaluation pooled results. The funnel plot in Figure S1, Figure S3 and Figure S4 also presented a certain degree of symmetry, indicating there was no potential for publication bias among the studies included in this analysis.

### Overall Diagnostic Performance of Fetal Echocardiography

#### STIC

**Figure 2:**
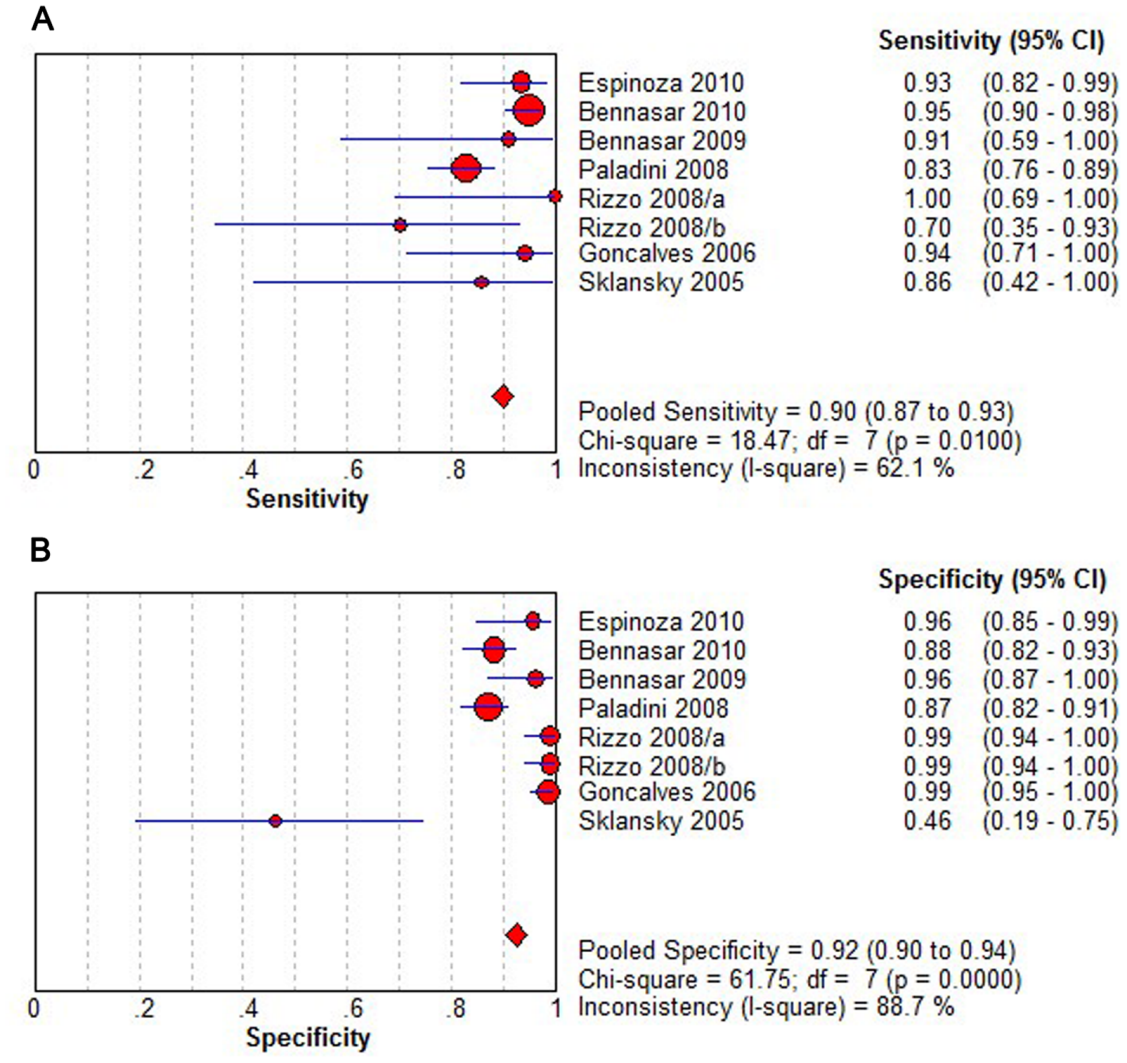
Sensitivity and specificity of STIC detection for the diagnosis of fetal CHDs. (A) Pooled sensitivity. (B) Pooled specificity. Effect sizes were pooled by random-effects models. The point estimates from each study are shown as solid squares. The pooled estimates are shown as a solid diamond. Error bars represent 95% CIs. STIC, spatiotemporal image correlation; CI, confidence interval; df, degrees of freedom.

**Figure 3:**
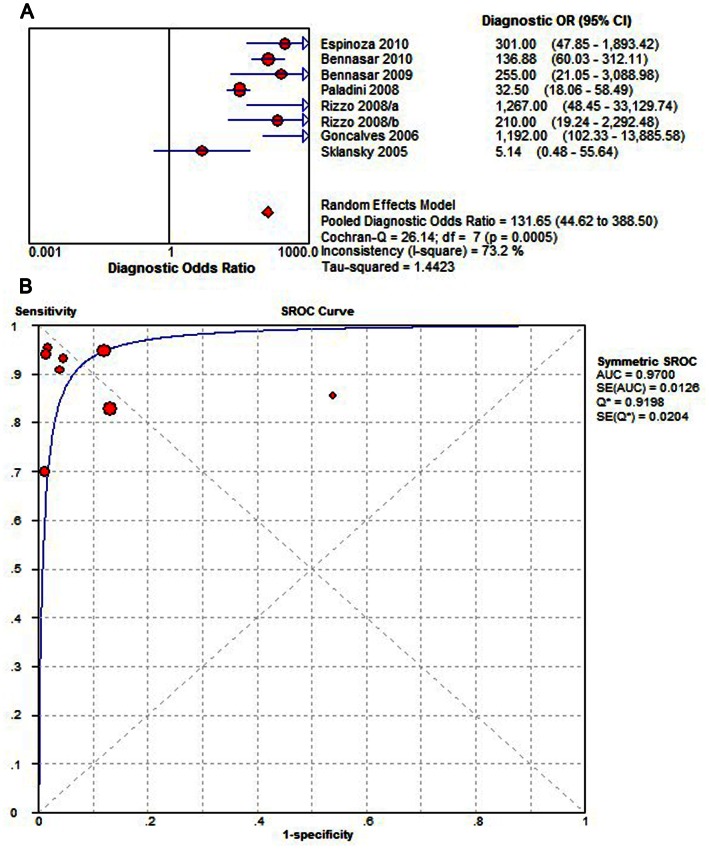
Overall diagnostic odds ratio and summary receiver operating characteristic curves for all data sets describing the diagnostic performance of STIC detection in identifying fetal CHDs. (A) Overall diagnostic odds ratio. (B) The summary receiver operating characteristic curves for all data sets. Effect sizes were pooled by random-effects models. The pooled diagnostic odds ratio is shown as a solid diamond. Each square in the summary receiver operating characteristic curve represents one study. Sample size is indicated by the size of the square. STIC, spatiotemporal image correlation; CI, confidence interval; df, degrees of freedom; DOR, diagnostic odds ratio; AUC, area under curve.

Overall diagnostic performance of STIC (Figure 2 and 3) shows the capability of STIC in detecting fetal CHD. The summary sensitivity was 0.90 (95% CI, 0.87 to 0.93), with individual sensitivities ranging from 0.70 to 1.00. The summary specificity was 0.92 (95% CI, 0.90 to 0.94), with individual specificities ranging from 0.46 to 0.99. Both pooled estimations showed significant heterogeneity (Sensitivity: P = 0.0100, X^2^ = 18.47, I^2^ = 62.1%; specificity: P = 0.0000, X^2^ = 61.75, I^2^ = 88.7%). The pooled diagnostic odds ratio was 131.65 (95% CI, 44.62 to 388.50), with individual diagnostic odds ratio s ranging from 5.14 to 1267.00. The results of diagnostic odds ratio showed no consistency across the included reports, with noticeable heterogeneity (P = 0.0005, Cochran-Q = 26.14, I^2^ = 73.2%). The point size in the summary receiver operating characteristic curve represented the proportional study weight. Most data gathered near the top left corner where sensitivity and specificity were both the highest. The the area under the curve value was 0.9700±0.0126. The absence of curvilinear shape in the summary receiver operating characteristic curve suggested no potential presence of a threshold effect.

#### ECEE

Overall Diagnostic Performance of ECEE shows the capability of ECEE in detecting fetal CHD. The summary sensitivity was 0.89 (95% CI, 0.87 to 0.90), with individual sensitivities ranging from 0.43 to 1.00. The summary specificity was 1.00 (95% CI, 1.00 to 1.00), with individual specificities ranging from 0.96 to 1.00. Both pooled estimations showed significant heterogeneity (Sensitivity: P = 0.0000, X^2^  = 168.03, I^2^ = 86.3%; specificity: P = 0.0000, X^2^  = 144.48, I^2^ = 84.1%). The pooled diagnostic odds ratio was 2538.16 (95% CI, 1144.50 to 5628.88), with individual diagnostic odds ratios ranging from 42.50 to 374862.84. The results of diagnostic odds ratio showed no consistency across the included reports, with noticeable heterogeneity (P = 0.0000, Cochran-Q = 77.38, I^2^ = 70.3%). The point size in the summary receiver operating characteristic curve represented the proportional study weight. Most data gathered near the top left corner where sensitivity and specificity were both the highest. The area under the curve value was 0.9971±0.0009. The absence of curvilinear shape in the summary receiver operating characteristic curve suggested no potential presence of a threshold effect.

#### 4 CV+OTV+3 VTV

**Figure 4:**
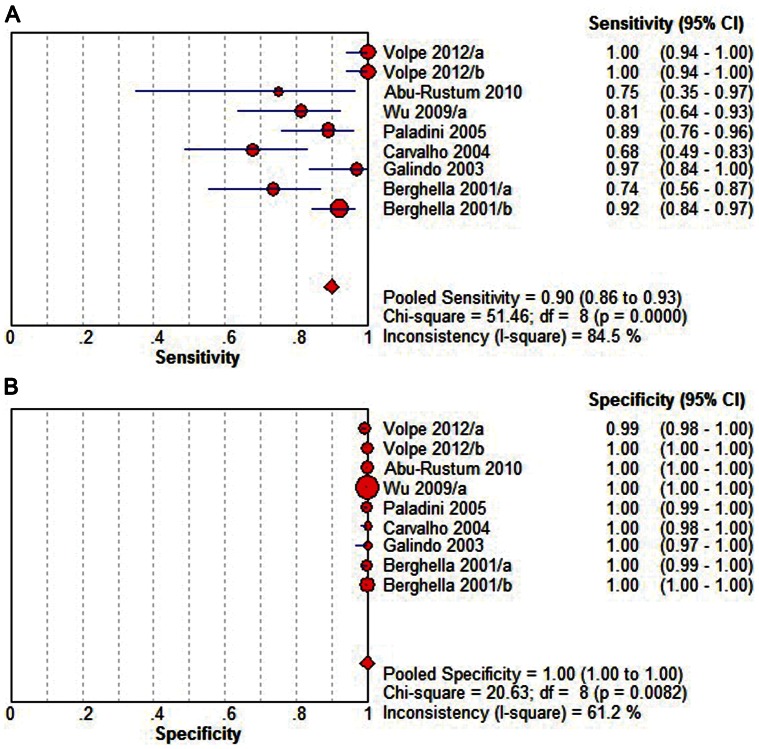
Sensitivity and specificity of 4 **CV+OTV+3** **VTV detection for the diagnosis of fetal CHDs.** (A) Pooled sensitivity. (B) Pooled specificity. Effect sizes were pooled by random-effects models. The point estimates from each study are shown as solid squares. The pooled estimates are shown as a solid diamond. Error bars represent 95% CIs. 4 CV, 4 chamber view; OTV, outflow tract view; VTV, three-vessel trachea view; CI, confidence interval; df, degrees of freedom.

Overall Diagnostic Performance of 4 CV+OTV+3 VTV (Figure 4) shows the capability of 4 CV+OTV+3 VTV in detecting fetal CHD. The summary sensitivity was 0.90 (95% CI, 0.86 to 0.93), with individual sensitivities ranging from 0.68 to 1.00. The summary specificity was 1.00 (95% CI, 1.00 to 1.00), with individual specificities ranging from 0.99 to 1.00. Both pooled estimations showed significant heterogeneity (Sensitivity: P = 0.0000, X^2^ = 51.46, I^2^ = 84.5%; specificity: P = 0.0082, X^2^ = 20.63, I^2^ = 61.2%). The pooled diagnostic odds ratio was 5224.27 (95% CI, 2071.12 to 13177.88), with individual diagnostic odds ratios ranging from 809.72 to 202125.00. The results of diagnostic odds ratio showed consistency across the included reports, without noticeable heterogeneity (P = 0.1188, Cochran-Q = 12.80, I^2^ = 37.5%). The point size in the summary receiver operating characteristic curve represented the proportional study weight. Most data gathered near the top left corner where sensitivity and specificity were both the highest. The area under the curve value was 0.9983±0.0008. The absence of curvilinear shape in the summary receiver operating characteristic curve suggested no potential presence of a threshold effect.

#### 4 CV+OTV/3 VTV

Overall Diagnostic Performance of 4 CV+OTV or 4 CV+3 VTV shows the capability of 4 CV+OTV or 4 CV+3 VTV in detecting fetal CHD. The summary sensitivity was 0.65 (95% CI, 0.61 to 0.69), with individual sensitivities ranging from 0.14 to 0.93. The summary specificity was 1.00 (95% CI, 1.00 to 1.00), with individual specificities ranging from 0.98 to 1.00. Both pooled estimations showed significant heterogeneity (Sensitivity: P = 0.0000, X^2^  = 68.44, I^2^ = 82.5%; specificity: P = 0.0000, X^2^  = 144.48, I^2^ = 91.7%). The pooled diagnostic odds ratio was 817.72 (95% CI, 310.54 to 2153.26), with individual diagnostic odds ratios ranging from 15.42 to 43402.38. The results of diagnostic odds ratio showed no consistency across the included reports, with noticeable heterogeneity (P = 0.0000, Cochran-Q = 76.17, I^2^ = 84.2%). The point size in the summary receiver operating characteristic curve represented the proportional study weight. Most data gathered near the left border where sensitivity diffused with a large range and specificity was the highest. The area under the curve value was 0.9929±0.0029. The absence of curvilinear shape in the summary receiver operating characteristic curve suggested no potential presence of a threshold effect.

#### 4 CV

Overall Diagnostic Performance of 4 CV shows the capability of 4 CV in detecting fetal CHD. The summary sensitivity was 0.52 (95% CI, 0.50 to 0.55), with individual sensitivities ranging from 0.15 to 1.00. The summary specificity was 1.00 (95% CI, 1.00 to 1.00), with individual specificities ranging from 0.94 to 1.00. Both pooled estimations showed significant heterogeneity (Sensitivity: P = 0.0000, X^2^  = 589.26, I^2^ = 96.1%; specificity: P = 0.0000, X^2^  = 252.76, I^2^ = 90.9%). The pooled diagnostic odds ratio was 804.37 (95% CI, 385.59 to 1677.95), with individual diagnostic odds ratios ranging from 50.19 to 43435.59. The results of diagnostic odds ratio showed no consistency across the included reports, with noticeable heterogeneity (P = 0.0000, Cochran-Q = 105.52, I^2^ = 78.2%). The point size in the summary receiver operating characteristic curve represented the proportional study weight. Most data gathered near the left border where sensitivity diffused with a large range and specificity was the highest. The area under the curve value was 0.9928±0.0022. The absence of curvilinear shape in the summary receiver operating characteristic curve suggested no potential presence of a threshold effect.

### Sensitivity Analysis

We systematically removed one data set at a time and recalculated the diagnostic odds ratio and area under the curve values for the remaining studies. These results indicated that no single data set carried enough weight to significantly influence the pooled test performance reported for the ability of each type of fetal echocardiography to identify cases of fetal CHD. Finally sensitivity analysis had been done by a larger sample size subgroup analysis in the comparison which enrolled more than 5 studies, and every analysis confirmed in both direction and magnitude of statistical significance the findings of the overall analysis.

### Analysis of Variance

The comparison of sensitivity and specificity among different types of echocardiography had been done by X^2^ test. Among 5 groups, the sensitivities and specificities were not all same for pooled results. Moreover, the sensitivities of STIC, ECEE and 4 CV+OTV+3 VTV showed no significant difference by comparison. However, the results of 4 CV+OTV/3 VTV and 4 CV pooled estimations showed significant differences between each group, with a significant lower sensitivity, especially for the 4 CV. The specificity of STIC pooled estimations showed significant differences between each group by comparison, with a significant lower specificity. However, the results of ECEE, 4 CV+OTV+3 VTV, 4 CV+OTV/3 VTV and 4 CV pooled estimations showed significant differences between each group, with almost the same specificities (Table 2).

**Table 2:** Analysis of Variance.

	STIC	ECEE	4 CV+OTV+3 VTV	4 CV+OTV/3 VTV
Sensitivity^a^				
ECEE	0.651^c^	–	–	–
4 CV+OTV+3vVTV	1.000^c^	0.579^c^	–	–
4 CV+OTV/3 VTV	<0.001^d^	<0.001^d^	<0.001^d^	–
4 CV	<0.001^d^	<0.001^d^	<0.001^d^	<0.001^d^
Specificity^b^				
ECEE	<0.001^d^	–	–	–
4 CV+OTV+3 VTV	<0.001^d^	0.992^c^	–	–
4 CV+OTV/3 VTV	<0.001^d^	0.996^c^	0.989^c^	–
4CV	<0.001^d^	0.776^c^	1.000^c^	0.699^c^

a The sensitivities of 5 groups were not all the same by X^2^ test with a p value <0.05.

b The specificities of 5 groups were not all the same by X^2^ test with a p value <0.05.

c Without significant difference as p value ≥ 0.05.

d With significant difference as p value < 0.05.

STIC, spatiotemporal image correlation; ECEE, extended cardiac echography examination; 4 CV, 4 chamber view; OTV, outflow tract view; VTV, three-vessel trachea view.

## Discussion

This meta-analysis was restricted to the characteristics and accuracy of different protocols of fetal echocardiography scanning. Since the introduction of fetal echocardiography from 1980s, many studies have focused on its effectiveness of detecting fetal CHDs, and provided convincing evidence about its reliability and high scan quality [44], [50], [57], [124]. Antenatal detection of CHDs remains one of the most challenging issues of prenatal diagnosis. Fetal cardiac abnormalities can be scanned and diagnosed as early as 11 weeks’ gestation by experienced groups [125], although the widely recommended age for performing routine fetal echocardiography is 22–24 weeks It is also reasonable to put the scanning time forward to 12–20 gestation weeks for high-risk pregnancies [126], [127]. Considering the superiority of prenatal diagnosis in helping neonatal administration and even life saving, fetal echocardiography has been listed in routine obstetrics ultrasound to provide more fetal information for parents [128], [129]. The doctors can be informed clearly about the fetal heart function and the hemodynamics of fetal circulation. When the fetus meets restricted and harmful hemodynamics which could lead to abortion, her or his mother could receive immediately cesarean to terminate the continuous depravation of fetal condition [6], [130]–[132]. Regarding this point, it is important to make a definite and scientific diagnosis.

Currently, most of cardiac malformations can be found out with the help of fetal echocardiography. Although amount of studies demonstrated the sensitivities and specificities of STIC, ECEE, 4 CV+OTV+3 VTV, 4 CV+OTV/3 VTV and 4 CV scan protocols, but the results showed dissemination with large ranges. To our knowledge, this is the first meta-analysis focused on the accuracy of prenatal diagnosis of CHD using 5 different types of echocardiography and make comparison among the 5 protocols. Randall et al. had drawn a systematic review on routine fetal detection of CHD among unselected and low risk populations [133] and Rasiah et al focused on the accuracy of first-trimester ultrasound examination for detecting major CHD [134]. Even these 2 meta-analyses about the accuracy of fetal echocardiography have been done, but they only took specialized indications for enrolled articles and provided some strict evidence about fetal CHD detection. So this meta-analysis concentrated on the common used 5 scan protocols, and demonstrated some instruction for fetal ultrasound scan selection.

In this meta-analysis, we included 63 relevant studies with a total of 81 studies. Among the pooled diagnostic odds ratios, the STIC had the lowest diagnostic odds ratio of 131.65 (95% CI, 44.62 to 388.50). The areas under the curve of the summary receiver operating characteristic curves for all data sets were higher than 0.99 which demonstrated a quite high diagnostic accuracy. And the area under the curve of summary receiver operating characteristic of STIC was 0.9700±0.0126. These results represented a good diagnostic efficacy for every method in identifying fetal CHD, regardless of the sample origin and methodology variation. STIC technology has been incorporated by some groups into the management of fetuses at high risk of CHDs [9]. The use of STIC in the first trimester has been reported only in some very recent series. STIC technology offers other advantages such as access to virtual planes not available for direct visualization in 2D ultrasound and multiplanar reconstruction to view three orthogonal planes simultaneously [10], [31], [86], [135]. The navigation dot in multiplanar reconstruction provides positioning and orientation assistance to the operator. There are functional cardiology analyses that can only be performed with STIC technology. Vinals et al. demonstrated that volume datasets from a first-trimester fetal heart can be acquired in a high proportion of cases by properly trained non-expert operators and sent to an expert in ECEE for offline evaluation via telemedicine [136]. Although non-experts in echocardiography could acquire correct volumes in all patients in Bennasar et al. series [78]. Though STIC technology has above advantages, it can not take all the place of the 2D ultrasound scan for its poorer specificity. As previously reported, there are some areas of difficulty in diagnosis of CHD, especially at 11 to 14 weeks. This difficulty applies particularly to minor defects, such as ventricular septal defects [83], [121], and to several forms of structural heart disease, which evolve in uterine and become apparent with the advancing of gestation.

To investigate potential variables of sensitivities and specificities among 5 scan protocols, a Χ^2^ analysis was conducted to provide clues for methodological indications. It found that the sensitivities had been stabled at a level about 0.90, which suggested that completed 3 sections view could provide a satisfied sensitivity. Even though more sections scan could provide more information about fetal heart, but to routine fetal heart examination for low risk fetuses, the sections viewed after finishing 4 CV, OTV and 3 VTV with high quality images can get a stable accurate diagnosis level, and may not shrink the accuracy. However, once the fetus had been identified CHD, the ECEE and STIC maybe helpful in supplying more information, especially for complex CHDs. But the new technology of STIC could not get a top performance of specificity which traditional 2D ultrasound showed almost no false positive. At the same time, these results suggested the STIC technique can not be a final diagnostic method for fetal CHD alone. 2D ultrasound should be performed firstly and consider the STIC as an additional examination to provide local detail information of defects.

For such fetus in the early term of gestation, there are some difficulties to obtain 3 cardiac sections or complete a whole ECEE examination [125], [137]. In this circumstances, it’s not responsible to make diagnosis of whether this fetus suffering from CHD. Longer term follow-up is still needed until echocardiography can be finished with more than 3 cardiac sections, especially for the pregnant woman with high risk factors. After that, the observers can make a scientific diagnosis and get more stereoscopic images for fetal evaluation or even fetal treatment, such as fetal cardiac intervention and neonatal surgery at the very beginning of life.

The limitations of this meta-analysis are: 1) only English publications were included; 2) univariate analysis about the examination weeks, with or without high risk and the publication years had not been done for the large heterogeneity. The potential influence factors analysis might get unconvinced results for few studies respectively.

In conclusion, despite inter-study variability, the test performance of fetal CHD detected by echocardiography technology was impressive and non-consistent under circumstances of methodological changes. But each method demonstrated both acceptable sensitivity and specificity in detecting fetal heart defects. These results suggest a great diagnostic potential for fetal echocardiography detection as a reliable method of fetal CHD. At least 3 sections view (4 CV, OTV and 3 VTV) should be included in routine scan protocols, but in the specific examination of fetal heart structure, the ECEE should be done for more range of imformation and it encourages that ECEE should be performaned for every high-risk pregnant women and in tertiary medical center. So that without 3 section view completed in primary scan, diagnosis of CHD can not be reached. While the STIC technology can be used to provide more detail information for local situation of defects, especailly for such fetus who would undergo fetal cardiac intervention, STIC may be quite helpful and provide exact instructions. However, STIC can not be used to make a definite diagnosis alone with its relatively low specificity.

## Supporting Information

Figure S1 **Funnel plot for the assessment of potential publication bias of STIC.** The funnel graphs plot the square root of the effective sample size (1/ESS1/2) against the diagnostic odds ratio. Each circle represents each study in the meta-analysis. Asymmetry of the circle distribution between regression lines indicates potential publication bias. This funnel plot indicates no publication bias with a p value = 0.28 > 0.10. ESS, effective sample size.
(TIF)

Figure S2 **Funnel plot for the assessment of potential publication bias of ECEE.** The funnel graphs plot the square root of the effective sample size (1/ESS1/2) against the diagnostic odds ratio. Each circle represents each study in the meta-analysis. Asymmetry of the circle distribution between regression lines indicates potential publication bias. This funnel plot indicates publication bias with a p value = 0.01 < 0.10. ESS, effective sample size.
(TIF)

Figure S3 **Funnel plot for the assessment of potential publication bias of 4 CV+OTV+3** **VTV.** The funnel graphs plot the square root of the effective sample size (1/ESS1/2) against the diagnostic odds ratio. Each circle represents each study in the meta-analysis. Asymmetry of the circle distribution between regression lines indicates potential publication bias. This funnel plot indicates no publication bias with a p value = 0.21 > 0.10. ESS, effective sample size.
(TIF)

Figure S4 **Funnel plot for the assessment of potential publication bias of 4** **CV+OTV/3** **VTV.** The funnel graphs plot the square root of the effective sample size (1/ESS1/2) against the diagnostic odds ratio. Each circle represents each study in the meta-analysis. Asymmetry of the circle distribution between regression lines indicates potential publication bias. This funnel plot indicates no publication bias with a p value = 0.15 > 0.10. ESS, effective sample size.
(TIF)

Figure S5 **Funnel plot for the assessment of potential publication bias of 4** **CV.** The funnel graphs plot the square root of the effective sample size (1/ESS1/2) against the diagnostic odds ratio. Each circle represents each study in the meta-analysis. Asymmetry of the circle distribution between regression lines indicates potential publication bias. This funnel plot indicates publication bias with a p value = 0.00 < 0.10. ESS, effective sample size.
(TIF)

Table S1 PRISMA 2009 check list.
(PDF)

Table S2 Quality assessment of the included articles. QUADAS, Quality Assessment of Diagnostic Accuracy Studies.
(DOC)
