# Supplementary figures and images for: Performance of Different Scan Protocols of Fetal Echocardiography in the Diagnosis of Fetal Congenital Heart Disease: A Systematic Review and Meta-Analysis
Source: PLoS One. 2013 Jun 4;8(6):e65484. doi: 10.1371/journal.pone.0065484 (PMC3672155; doi:10.1371/journal.pone.0065484)

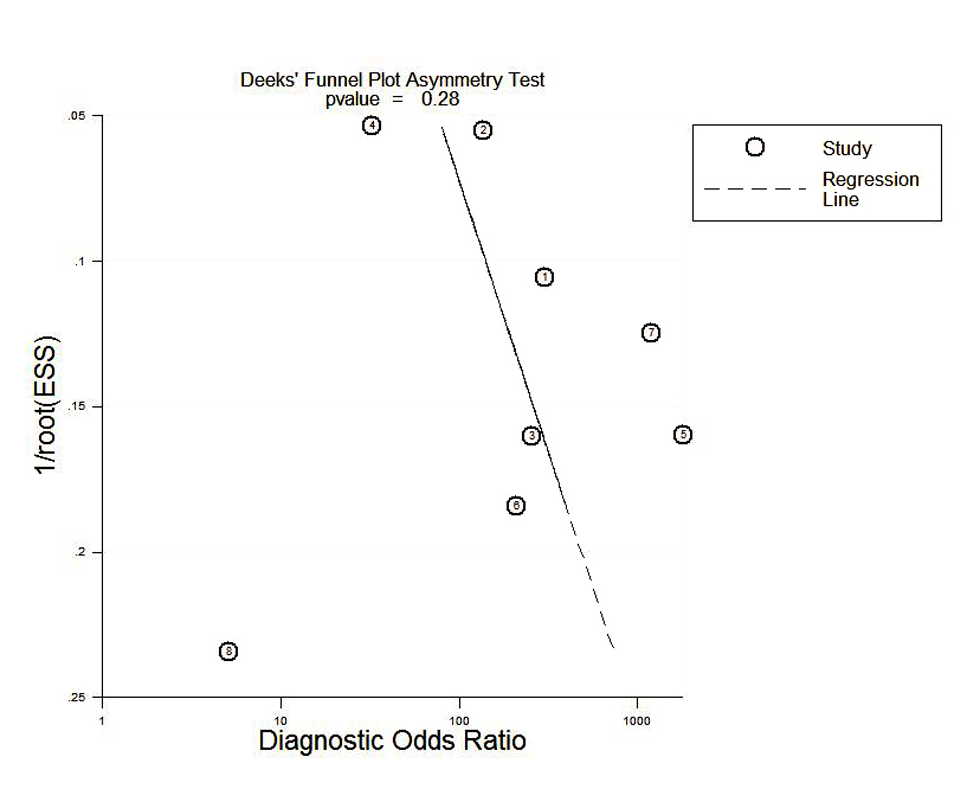

Supplement: Figure S1 — Funnel plot for the assessment of potential publication bias of STIC. The funnel graphs plot the square root of the effective sample size (1/ESS1/2) against the diagnostic odds ratio. Each circle represents each study in the meta-analysis. Asymmetry of the circle distribution between regression lines indicates potential publication bias. This funnel plot indicates no publication bias with a p value = 0.28 > 0.10. ESS, effective sample size. (TIF) [file pone.0065484.s001.tif]

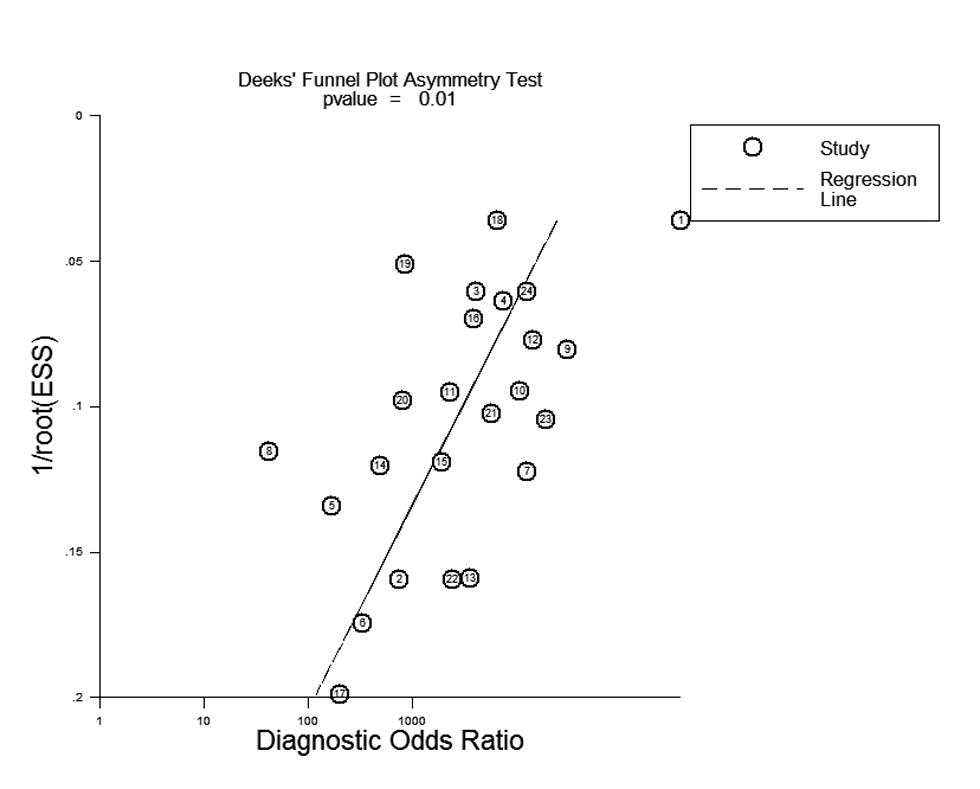

Supplement: Figure S2 — Funnel plot for the assessment of potential publication bias of ECEE. The funnel graphs plot the square root of the effective sample size (1/ESS1/2) against the diagnostic odds ratio. Each circle represents each study in the meta-analysis. Asymmetry of the circle distribution between regression lines indicates potential publication bias. This funnel plot indicates publication bias with a p value = 0.01 < 0.10. ESS, effective sample size. (TIF) [file pone.0065484.s002.tif]

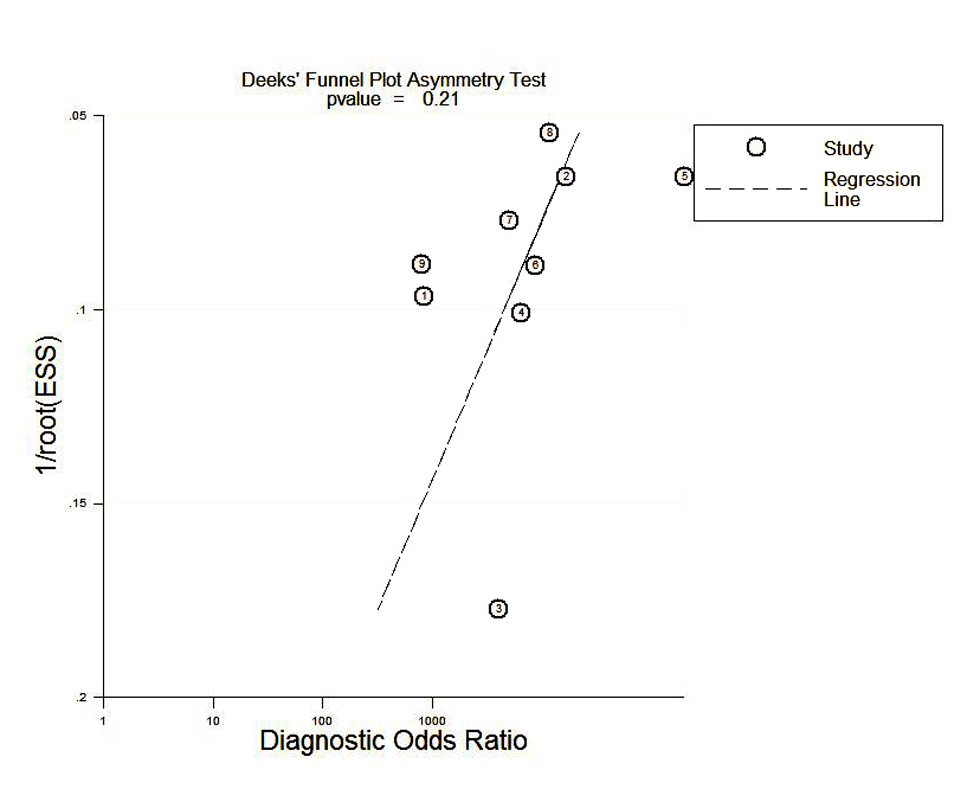

Supplement: Figure S3 — Funnel plot for the assessment of potential publication bias of 4 CV+OTV+3 VTV. The funnel graphs plot the square root of the effective sample size (1/ESS1/2) against the diagnostic odds ratio. Each circle represents each study in the meta-analysis. Asymmetry of the circle distribution between regression lines indicates potential publication bias. This funnel plot indicates no publication bias with a p value = 0.21 > 0.10. ESS, effective sample size. (TIF) [file pone.0065484.s003.tif]

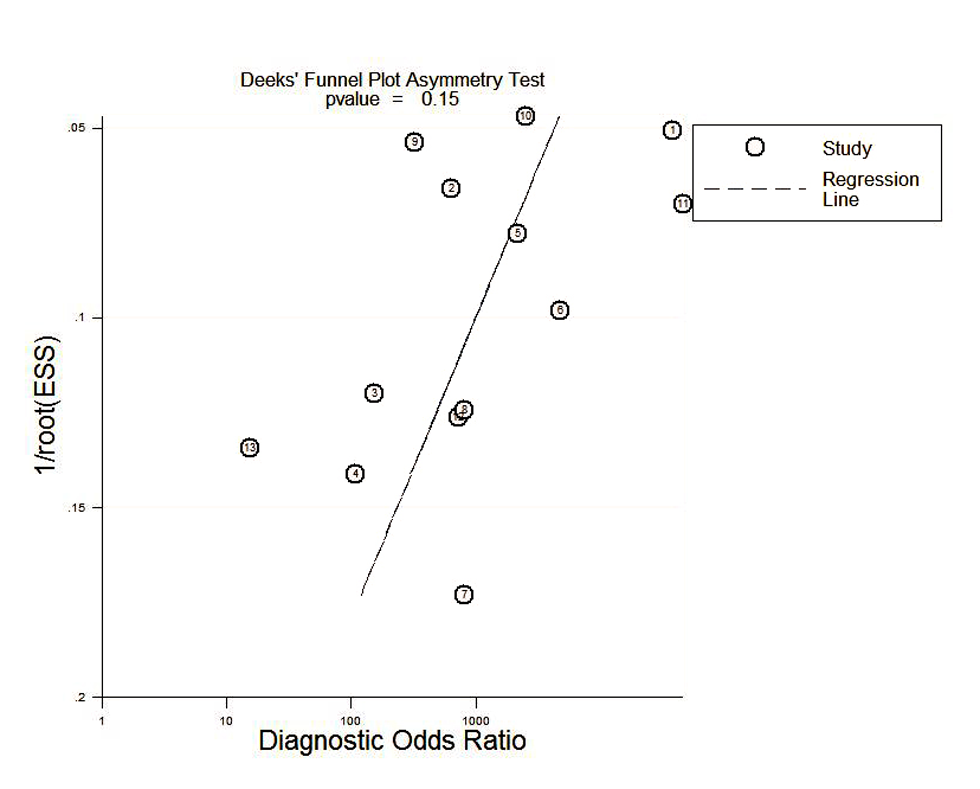

Supplement: Figure S4 — Funnel plot for the assessment of potential publication bias of 4 CV+OTV/3 VTV. The funnel graphs plot the square root of the effective sample size (1/ESS1/2) against the diagnostic odds ratio. Each circle represents each study in the meta-analysis. Asymmetry of the circle distribution between regression lines indicates potential publication bias. This funnel plot indicates no publication bias with a p value = 0.15 > 0.10. ESS, effective sample size. (TIF) [file pone.0065484.s004.tif]

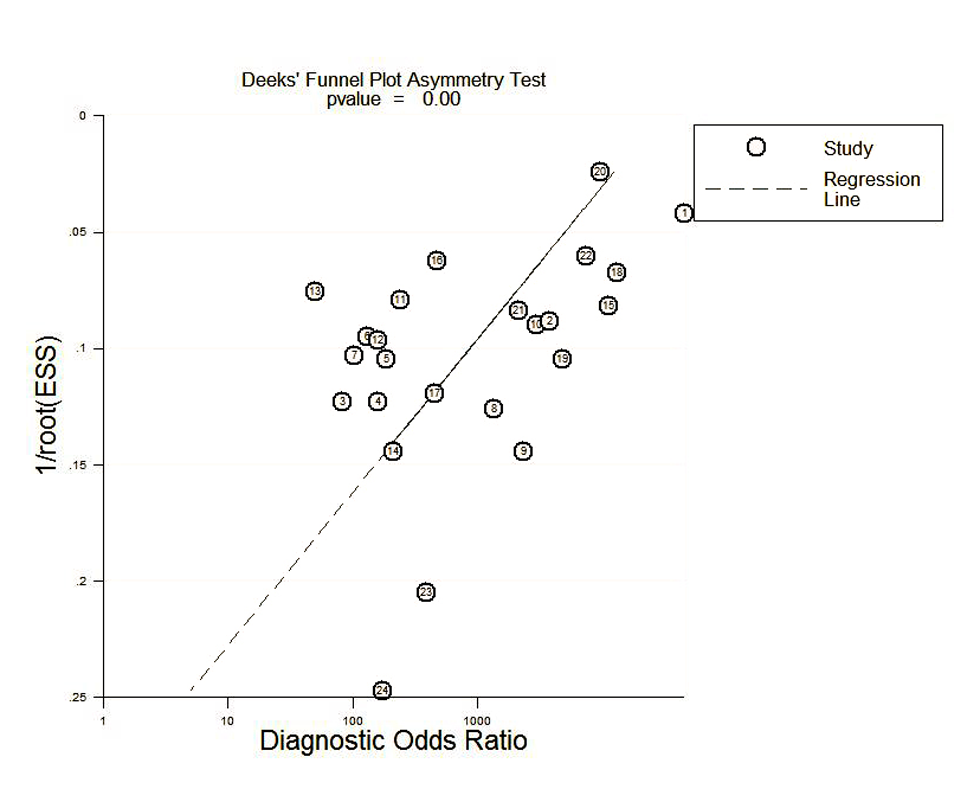

Supplement: Figure S5 — Funnel plot for the assessment of potential publication bias of 4 CV. The funnel graphs plot the square root of the effective sample size (1/ESS1/2) against the diagnostic odds ratio. Each circle represents each study in the meta-analysis. Asymmetry of the circle distribution between regression lines indicates potential publication bias. This funnel plot indicates publication bias with a p value = 0.00 < 0.10. ESS, effective sample size. (TIF) [file pone.0065484.s005.tif]
