# Supplementary material for: Performance of Different Scan Protocols of Fetal Echocardiography in the Diagnosis of Fetal Congenital Heart Disease: A Systematic Review and Meta-Analysis
Source: PLoS One. 2013 Jun 4;8(6):e65484. doi: 10.1371/journal.pone.0065484 (PMC3672155; doi:10.1371/journal.pone.0065484)
Supplement: Table S2 — Quality assessment of the included articles. QUADAS, Quality Assessment of Diagnostic Accuracy Studies. (DOC) [file pone.0065484.s007.doc]

**Table S**2 QUADAS criteria of included studies

| **No.** | **Spectrum composition** | **Selection criteria** | **Reference standard** | **Disease progression bias** | **Partial verification** | **Differential verification** | **Incorporation bias** | **Index test execution** | **Reference standard execution** | **Test review bias** | **Reference standard review bias** | **Clinical review bias** | **Uninterruptible test results** | **Withdrawals** |
| --- | --- | --- | --- | --- | --- | --- | --- | --- | --- | --- | --- | --- | --- | --- |
| **1** | **+** | **+** | **+** | **?** | **+** | **+** | **+** | **+** | **+** | **+** | **?** | **+** | **+** | **+** |
| **2** | **+** | **+** | **+** | **+** | **+** | **+** | **+** | **+** | **+** | **+** | **?** | **+** | **+** | **+** |
| **3** | **+** | **+** | **+** | **+** | **+** | **-** | **+** | **+** | **+** | **+** | **?** | **+** | **+** | **+** |
| **4** | **+** | **+** | **+** | **+** | **+** | **+** | **+** | **+** | **+** | **+** | **?** | **+** | **+** | **?** |
| **5** | **+** | **+** | **+** | **+** | **+** | **+** | **+** | **+** | **+** | **+** | **+** | **+** | **+** | **+** |
| **6** | **+** | **+** | **+** | **+** | **+** | **+** | **+** | **+** | **?** | **+** | **?** | **+** | **+** | **+** |
| **7** | **+** | **+** | **+** | **+** | **+** | **+** | **+** | **+** | **?** | **+** | **?** | **+** | **+** | **?** |
| **8** | **+** | **+** | **+** | **+** | **+** | **+** | **+** | **+** | **+** | **+** | **-** | **+** | **+** | **+** |
| **9** | **+** | **+** | **+** | **+** | **+** | **+** | **+** | **+** | **+** | **+** | **?** | **+** | **+** | **+** |
| **10** | **+** | **+** | **+** | **+** | **+** | **+** | **+** | **+** | **+** | **+** | **?** | **+** | **+** | **+** |
| **11** | **+** | **+** | **+** | **+** | **+** | **+** | **+** | **+** | **+** | **+** | **?** | **+** | **+** | **+** |
| **12** | **+** | **+** | **+** | **+** | **+** | **+** | **+** | **?** | **+** | **+** | **?** | **+** | **+** | **+** |
| **13** | **+** | **+** | **+** | **+** | **+** | **+** | **+** | **?** | **+** | **+** | **?** | **+** | **+** | **+** |
| **14** | **+** | **+** | **+** | **+** | **+** | **+** | **+** | **+** | **+** | **+** | **-** | **+** | **+** | **+** |
| **15** | **+** | **?** | **+** | **+** | **+** | **+** | **+** | **+** | **+** | **+** | **?** | **+** | **+** | **+** |
| **16** | **+** | **+** | **+** | **+** | **+** | **+** | **+** | **+** | **+** | **+** | **?** | **+** | **+** | **+** |
| **17** | **+** | **+** | **+** | **+** | **+** | **+** | **+** | **+** | **+** | **+** | **?** | **+** | **+** | **+** |
| **18** | **+** | **+** | **+** | **+** | **+** | **+** | **+** | **+** | **+** | **+** | **?** | **+** | **+** | **+** |
| **19** | **+** | **+** | **+** | **+** | **+** | **+** | **+** | **+** | **+** | **+** | **?** | **+** | **+** | **+** |
| **20** | **+** | **+** | **+** | **+** | **+** | **+** | **+** | **+** | **+** | **+** | **-** | **+** | **+** | **+** |
| **21** | **+** | **+** | **+** | **+** | **+** | **+** | **+** | **?** | **+** | **+** | **+** | **+** | **+** | **+** |
| **22** | **+** | **+** | **+** | **+** | **+** | **+** | **+** | **+** | **+** | **+** | **?** | **+** | **+** | **+** |
| **23** | **+** | **+** | **?** | **+** | **+** | **+** | **+** | **?** | **+** | **+** | **+** | **+** | **?** | **+** |
| **24** | **+** | **+** | **+** | **+** | **+** | **+** | **+** | **?** | **+** | **+** | **?** | **+** | **+** | **+** |
| **25** | **+** | **+** | **+** | **+** | **+** | **+** | **+** | **+** | **+** | **+** | **?** | **+** | **+** | **+** |
| **26** | **+** | **+** | **+** | **+** | **+** | **+** | **+** | **+** | **+** | **+** | **-** | **+** | **+** | **+** |
| **27** | **+** | **+** | **+** | **+** | **+** | **+** | **+** | **+** | **+** | **+** | **?** | **+** | **?** | **+** |
| **28** | **+** | **+** | **+** | **+** | **+** | **+** | **+** | **+** | **+** | **+** | **?** | **+** | **+** | **+** |
| **29** | **+** | **+** | **+** | **+** | **+** | **-** | **+** | **+** | **+** | **+** | **?** | **+** | **+** | **?** |
| **30** | **+** | **+** | **+** | **+** | **+** | **+** | **+** | **+** | **+** | **+** | **+** | **+** | **+** | **+** |
| **31** | **+** | **+** | **+** | **+** | **+** | **+** | **+** | **+** | **+** | **+** | **?** | **+** | **+** | **+** |
| **32** | **+** | **+** | **+** | **+** | **+** | **-** | **+** | **+** | **+** | **+** | **?** | **+** | **+** | **+** |
| **33** | **+** | **+** | **+** | **+** | **+** | **+** | **+** | **+** | **+** | **+** | **?** | **+** | **+** | **+** |
| **34** | **+** | **+** | **+** | **+** | **+** | **+** | **+** | **+** | **+** | **+** | **?** | **+** | **+** | **+** |
| **35** | **+** | **+** | **+** | **+** | **+** | **+** | **+** | **+** | **+** | **+** | **?** | **+** | **+** | **+** |
| **36** | **+** | **+** | **+** | **+** | **+** | **+** | **+** | **+** | **+** | **+** | **?** | **+** | **+** | **+** |
| **37** | **+** | **+** | **+** | **+** | **+** | **+** | **+** | **+** | **+** | **+** | **-** | **+** | **+** | **+** |
| **38** | **+** | **+** | **+** | **+** | **+** | **?** | **+** | **+** | **+** | **+** | **?** | **+** | **+** | **+** |
| **39** | **+** | **+** | **+** | **+** | **+** | **+** | **+** | **+** | **+** | **+** | **?** | **+** | **+** | **+** |
| **40** | **+** | **+** | **+** | **+** | **+** | **+** | **+** | **?** | **+** | **+** | **-** | **+** | **+** | **+** |
| **41** | **+** | **+** | **+** | **+** | **+** | **+** | **+** | **+** | **+** | **+** | **?** | **+** | **+** | **+** |
| **42** | **+** | **+** | **+** | **+** | **+** | **+** | **+** | **+** | **+** | **+** | **?** | **+** | **+** | **+** |
| **43** | **+** | **+** | **+** | **+** | **+** | **+** | **+** | **+** | **+** | **+** | **?** | **+** | **+** | **+** |
| **44** | **+** | **+** | **+** | **+** | **+** | **+** | **+** | **+** | **+** | **+** | **?** | **+** | **+** | **+** |
| **45** | **+** | **+** | **+** | **+** | **+** | **+** | **+** | **+** | **+** | **+** | **?** | **+** | **+** | **?** |
| **46** | **+** | **+** | **+** | **+** | **+** | **+** | **+** | **+** | **+** | **+** | **?** | **+** | **+** | **+** |
| **47** | **+** | **+** | **+** | **+** | **+** | **+** | **+** | **+** | **+** | **+** | **?** | **+** | **+** | **+** |
| **48** | **+** | **+** | **+** | **+** | **+** | **+** | **+** | **+** | **+** | **+** | **+** | **+** | **+** | **+** |
| **49** | **+** | **+** | **+** | **+** | **+** | **+** | **+** | **+** | **+** | **+** | **?** | **+** | **+** | **+** |
| **50** | **+** | **+** | **+** | **+** | **+** | **+** | **+** | **+** | **+** | **+** | **?** | **+** | **+** | **+** |
| **51** | **+** | **+** | **+** | **+** | **+** | **+** | **+** | **+** | **+** | **+** | **?** | **+** | **+** | **+** |
| **52** | **+** | **+** | **+** | **+** | **+** | **+** | **+** | **+** | **+** | **+** | **?** | **+** | **+** | **+** |
| **53** | **+** | **+** | **+** | **+** | **+** | **+** | **+** | **+** | **+** | **+** | **?** | **+** | **+** | **+** |
| **54** | **+** | **+** | **+** | **+** | **+** | **+** | **+** | **+** | **+** | **+** | **?** | **+** | **+** | **+** |
| **55** | **+** | **+** | **+** | **+** | **+** | **+** | **+** | **+** | **+** | **+** | **?** | **+** | **+** | **+** |
| **56** | **+** | **+** | **+** | **+** | **+** | **+** | **+** | **+** | **+** | **+** | **?** | **+** | **+** | **+** |
| **57** | **+** | **?** | **+** | **+** | **+** | **+** | **+** | **+** | **+** | **+** | **?** | **+** | **+** | **?** |
| **58** | **+** | **+** | **+** | **+** | **+** | **+** | **+** | **+** | **+** | **+** | **?** | **+** | **+** | **+** |
| **59** | **+** | **+** | **+** | **+** | **+** | **+** | **+** | **+** | **+** | **+** | **?** | **+** | **+** | **+** |
| **60** | **+** | **+** | **+** | **+** | **+** | **+** | **+** | **+** | **+** | **+** | **?** | **+** | **+** | **+** |
| **61** | **+** | **+** | **+** | **+** | **+** | **+** | **+** | **+** | **+** | **+** | **?** | **+** | **+** | **+** |
| **62** | **+** | **+** | **+** | **+** | **+** | **+** | **+** | **+** | **+** | **+** | **?** | **+** | **+** | **+** |
| **63** | **+** | **+** | **+** | **+** | **+** | **+** | **+** | **+** | **+** | **+** | **?** | **+** | **+** | **+** |
